# Nisin, an apoptogenic bacteriocin and food preservative, attenuates HNSCC tumorigenesis via CHAC1

**DOI:** 10.1002/cam4.35

**Published:** 2012-10-02

**Authors:** Nam E Joo, Kathryn Ritchie, Pachiyappan Kamarajan, Di Miao, Yvonne L Kapila

**Affiliations:** Department of Periodontics and Oral Medicine, University of Michigan School of DentistryAnn Arbor, Michigan

**Keywords:** Cancer biology, cellular biology, drug discovery and delivery, translational research

## Abstract

Nisin, a bacteriocin and commonly used food preservative, may serve as a novel potential therapeutic for treating head and neck squamous cell carcinoma (HNSCC), as it induces preferential apoptosis, cell cycle arrest, and reduces cell proliferation in HNSCC cells, compared with primary keratinocytes. Nisin also reduces HNSCC tumorigenesis in vivo. Mechanistically, nisin exerts these effects on HNSCC, in part, through CHAC1, a proapoptotic cation transport regulator, and through a concomitant CHAC1-independent influx of extracellular calcium. In addition, although CHAC1 is known as an apoptotic mediator, its effects on cancer cell apoptosis have not been examined. Our studies are the first to report CHAC1's new role in promoting cancer cell apoptosis under nisin treatment. These data support the concept that nisin decreases HNSCC tumorigenesis in vitro and in vivo by inducing increased cell apoptosis and decreased cell proliferation; effects that are mediated by activation of CHAC1, increased calcium influxes, and induction of cell cycle arrest. These findings support the use of nisin as a potentially novel therapeutic for HNSCC, and as nisin is safe for human consumption and currently used in food preservation, its translation into a clinical setting may be facilitated.

## Introduction

Oral cancer is a leading cause of death worldwide, and oral squamous cell carcinoma (OSCC) accounts for more than 90% of oral malignancies (http://seer.cancer.gov/statfacts), yet survival rates for oral cancer have not improved in decades. These disheartening statistics underscore the need to examine its pathogenesis to help identify novel biomarkers and modes of therapy.

This study investigated the potential use of antimicrobial peptides (bacteriocins) as antitumor agents. Many biological functions have been attributed to antimicrobial peptides, including inhibition of membrane protein synthesis, inhibition of DNA synthesis, antiviral properties, and antitumor effects, which include induction of apoptosis or cytotoxicity of tumor cells [1–3]. Because of these properties, antimicrobial peptides have been investigated as potential therapeutic drugs [4].

Our data indicate that a commonly used food preservative and bacteriocin, nisin, has antitumor potential. Nisin is a 34-amino acid polycyclic antibacterial peptide that is produced by fermentation of the gram-positive bacterium *Lactococcus lactis*. Many antibacterial agents are effective against similar bacterial species; however, nisin has broad-spectrum effects, as it also inhibits gram-negative bacteria [5]. While bacteriocins, like nisin, have been used for years in preventing bacterial growth in foods, they have only recently been tested for prevention of growth of cancer cells. Nisin is not toxic to animals, is safe for human consumption, and was approved for human use by the WHO in 1969 and by the FDA in 1988. About 0.6 mg of nisin are consumed per person per day as part of normal food consumption (http://www.fda.gov/Food/FoodIngredientsPackaging/GenerallyRecognizedasSafeGRAS/GRASListings/ucm153977.htm). Therefore, development of nisin as a cancer therapeutic can be readily pursued following dosing determinations.

Nisin acts by altering the integrity of the cellular membrane and forming short-lived pores, thereby changing the membrane potential [6]. Nisin becomes immersed in the cell membrane through the cationic portions of the amino acids extending to one side of the molecule. These cationic portions interact with the negatively charged phospholipid heads, while the hydrophobic portion of nisin interacts with the membrane core [7]. Nisin's involvement with the membrane, therefore, mediates phospholipid reorganization and allows for an influx of ions [6, 8]. Nisin is known to preferentially interact with phosphatidylcholine [9]. As head and neck squamous cell carcinoma (HNSCC) cells and primary keratinocytes differ in their lipid membrane composition and function and response to calcium fluxes [10–15], nisin's ability to alter the transmembrane potential and membrane composition of cells may lead to differential effects on these cells. Indeed, our data support this premise as the basis for the nisin-mediated differential apoptotic cell death and reduced proliferation of HNSCC cells compared to primary keratinocytes.

Although the antibacterial properties of nisin have been used for decades in food preservation, it has only recently been examined in altering other forms of cell growth. Apoptosis or programmed cell death is a natural process that eliminates older cells. However, cancer cells are resistant to apoptosis, and thus, there is great effort to understand the mechanisms that regulate apoptosis in these cells. Mitochondria and endoplasmic reticulum begin the degradation process of apoptosis [16]. Released cytochrome c from the mitochondria binds to endoplasmic reticulum, causing the release of calcium. Calcium and cytochrome c release are key to apoptosome formation, and thereby to activation of caspases and nucleases, which cleave substrates and DNA to propagate apoptosis. Calcium is therefore a key regulatory ion not only in cell death but also in cell survival pathways. Calcium can also mediate apoptosis by altering the activation of cell surface death receptors and caspases. The role that calcium may play in nisin-triggered cell death has not been delineated. However, because nisin produces pores in cell membranes that lead to a net influx of ions, such as calcium, calcium may play a significant role in the induction of apoptosis and changes in cell proliferation in cells.

Given nisin's role as a safe bacteriocin for food preservation, and the knowledge that other bacteriocins possess proapoptotic properties against cancer cells, the goals of this study were to examine the effects and mechanisms of nisin on HNSCC cell apoptosis and cell proliferation in vitro and in vivo in an oral cancer mouse model. If effective, nisin could form the basis of a novel therapeutic for HNSCC.

## Methods

### Cell culture

Three human HNSCC cell lines and primary human oral keratinocytes were used for these studies. HNSCC cell line authentication and origin was provided by their sources and published extensively. The human HNSCC cell lines, UM-SCC-17B, and UM-SCC-14A were provided by Tom Carey (University of Michigan, MI). The oral SCC cell line HSC-3 was provided by Randall Kramer (University of California, San Francisco, CA). HNSCC cells were maintained in Dulbecco's modified Eagle's medium (DMEM) containing 10% fetal bovine serum, 1% penicillin, and 1% streptomycin. Primary human oral keratinocytes were purchased from Science Cell Research Laboratories and maintained in oral keratinocyte medium.

### Assessment of intracellular calcium concentration

Changes in intracellular calcium concentration mediated by nisin (Sigma, St. Louis, MO) and treatment with the calcium channel blocker, Bepridil (Sigma), were measured with the calcium sensitive dye Fluo-4 (Fluo-4 NW calcium assay kit, Invitrogen, Carlsbad, CA) in a multidetection microplate reader. After treatment with nisin and Bepridil, Bepridil alone, nisin alone, or no treatment, media were removed and replaced with 100 μL of Fluo-4 NW dye mix according to the manufacturer's protocol. The samples were excited at 494 nm and fluorescence emission was measured at 516 nm.

### Apoptosis assay

A DNA fragmentation ELISA (Roche Diagnostics, Indianapolis, IN) was used to assess cellular apoptosis following various treatments. Specifically, this assay used specific monoclonal antibodies directed against histones from fragmented DNA, allowing the determination of mono- and oligonucleosomes in the cytoplasmic fraction of cell lysates. Briefly, cells were plated in six-well culture plates overnight and treated with different concentrations of nisin for 24 h. Following this incubation period, the cells were lysed and assayed according the manufacturer's instructions. DNA fragmentation was evaluated by spectrophotometric analysis of samples within microtiter plates using a plate reader set at 405 nm, and the values were plotted as absorbance units.

### Cell cycle analyses

Following treatment with nisin or no treatment, cells were harvested and suspended in enzyme-free dissociation buffer (Invitrogen, Carlsbad, CA). Cells were then stained by addition of 250 μL of 50 μg/mL propidium iodide, 0.15% Triton X-100, and 150 μg/mL RNase A before cell cycle analysis by flow cytometry (Becton Dickinson, Franklin Lakes, NJ).

### Gene array analyses

To examine the mechanism regulating nisin-mediated actions on HNSCC cells, gene array analyses were performed on nisin treated (20 μg/mL of nisin overnight) and untreated HSC-3 cells. In all, 250 ng of total RNA was isolated from these cells using the RNeasy mini kit (Qiagen, Valencia, CA). Amplification and hybridization were performed by the University of Michigan Comprehensive Cancer Center Affymetrix and Microarray Core Facility using the GeneChip Mouse Genome 430 2.0 Array (Affymetrix, Santa Clara, CA). Approximately 39,000 genes were examined with the gene arrays, and differences greater than twofold in the nisin-treated groups compared with untreated controls were noted.

### CHAC1 suppression and overexpression

CHAC1 human-specific siRNAs (SASI_Hs01_00146246, SASI_Hs01_00146247, SASI_Hs01_00146249, SASI_Hs01_00146250, SASI_Hs01_00146251; and a scrambled control; Sigma) were transfected into HNSCC cells with HiPerFect reagent (Qiagen) according to manufacturer's protocols. For stable suppression of CHAC1, CHAC1 shRNA lentiviral particles (SHCLNG-NM_024111; TRCN0000154346, TRCN0000157127, TRCN0000157739, TRCN0000158316, and control shRNA; Sigma) were transduced into HNSCC cells then selected in 10 μg/mL puromycin for an additional 10 days (sc-108071; Santa Cruz Biotechnology, Santa Cruz, CA). Surviving cell colonies were picked and propagated before testing for CHAC1 expression. CHAC1 cDNA (provided by Dr. Imran Mungrue, UCLA [17]) and vector control were transfected into HNSCC cells using Lipofectamine 2000 (Invitrogren, Grand Island, NY) according to manufacturer's protocols. Following transfection/transduction, standard Western blotting was used to verify that suppression and overexpression of CHAC1 was achieved (CHAC1 antibody, AV42623, Sigma). Following efficient transfection, cells were treated with nisin or untreated, then changes in cell proliferation, apoptosis, and calcium influx levels were assessed.

### In vivo toxicity

The maximum tolerated drug doses are usually 57–75% of the LD50 dose in mice [18]. Early safety studies showed that oral administration of nisin in the range of 0.0125–125 μg/kg per day did not show negative side effects on growth and tissues in rats [19]. Other studies with higher doses of nisin (15–20 mg/kg per day) demonstrated that nisin did not have adverse effect on rats [20]. Based on the rat reproduction/chronic toxicity study by [21], the FDA and WHO accepted the highest dose tested in that study, 83.25 mg/kg of diet, as the no-observed-effect-level (NOEL) and affirmed the generally recognized as safe (GRAS) status of nisin. In this study, acute toxicity from daily administration of 150 mg/kg of nisin by oral gavage were monitored for 3 weeks in 3-week-old athymic nude mice (NCr-nu/nu strain, NCI, Frederick, MD). Mice were monitored daily and euthanized at the end of 3 weeks, at which time the major organs that metabolize drugs, the liver and kidney, were harvested and processed for histological examination.

### Oral cancer mouse model

To examine the effects of nisin, an oral cancer floor-of-mouth mouse model was used. HNSCC cells were injected submucosally into the floor of the mouth in mice as previously described [22, 23]. All protocols for in vivo studies were submitted for approval to the Committee on the Use and Care of Animals at the University of Michigan. Specifically, cells were grown to 70% confluence, suspended in DMEM, mixed with an equal volume of growth factor-reduced Matrigel basement membrane matrix (BD Biosciences, San Jose, CA) at a final concentration of 1.25 × 10^5^/0.05 mL. Six-week-old athymic nude mice (NCr-nu/nu strain, NCI, Frederick, MD) were anesthetized by intraperitoneal injection of 100 mg/kg ketamine and 10 mg/kg xylazine. A total volume of 0.05 mL of SCC cell/Matrigel suspension was injected submucosally into the floor of the mouth. Tumor volume was assessed weekly with digital calipers. Daily administration of nisin or water (equal volume/control) by oral gavage was tested using two nisin treatment regimens. A nisin pretreatment group, where nisin administration (200 mg/kg per day) was started 3 weeks before tumor cell injections and continued for 3 weeks thereafter, was used to examine whether preloading with nisin would be efficacious. The other treatment group consisted of nisin administration posttumor cell injection, where nisin (200 mg/kg per day) was administered 3 weeks after initial tumor cell injections (or after tumor cell growth was verified and palpable) and continued for 3 weeks thereafter. For CHAC1 suppression experiments, nisin (200 mg/kg per day) was given for 3 weeks following tumor cell injections. Following completion of nisin administration, mice were euthanized by CO_2_ overdose and cervical dislocation, and the primary tumor and kidney, heart, lungs, and liver were harvested, rinsed in PBS, and fixed overnight in 10% buffered formalin. Tissues were paraffin-embedded, sectioned, and processed for routine histopathological assessment with hematoxylin and eosin staining.

### Statistical analyses

In general, values are expressed as means ± SD. Intergroup differences were determined by two-way analysis of variance (ANOVA) and Scheffe's multiple-comparison test. Individual *P* values for each data set are indicated individually in each figure. For the in vivo studies, independent *t* tests with unequal variances were used. All experiments were repeated at least three times.

## Results

### Nisin increases apoptosis and reduces cell proliferation in HNSCC cells

**Figure 1:**
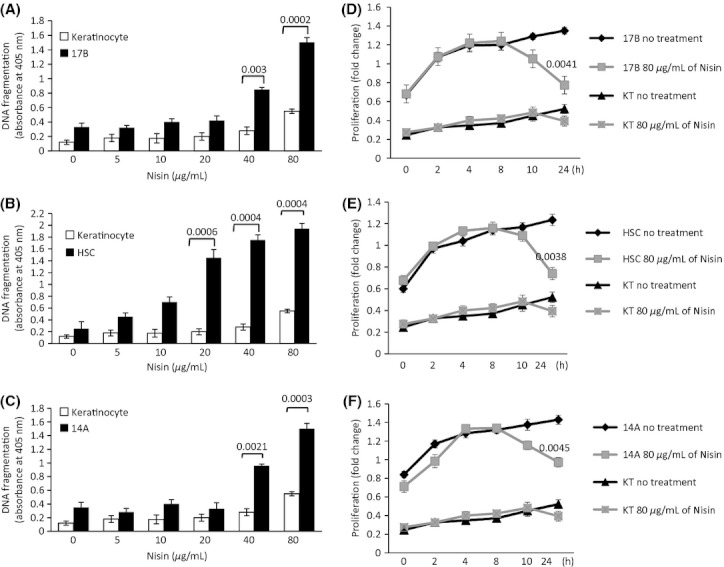
Nisin preferentially induces apoptosis and inhibits cell proliferation in head and neck squamous cell carcinoma (HNSCC) cells versus primary keratinocytes. (A–C) DNA fragmentation after 24 h and (D–F) fold change in cell proliferation in HNSCC cells (UM-SCC-17B, HSC-3, and UM-SCC-14A) after treatment with nisin as indicated. *P* values for each data set are indicated individually.

Treatment of three different HNSCC cell lines with increasing concentrations of nisin (5, 10, 20, 40, and 80 μg/mL) induced increased levels of DNA fragmentation or apoptosis after 24 h of treatment (Fig. 1). Significant increases in DNA fragmentation emerged in HNSCC cells when nisin concentrations reached over 20 μg/mL and up to 80 μg/mL. In contrast, primary oral keratinocytes did not exhibit elevated levels of DNA fragmentation like HNSCC cells. Nisin treatment with 80 μg/mL also reduced cell proliferation in three HNSCC cell lines over time with significant differences noted after 24 h of treatment. In contrast, primary oral keratinocytes did not exhibit decreases in cell proliferation over time upon treatment with the same concentration of nisin. Therefore, nisin preferentially increases DNA fragmentation or apoptosis and decreases cell proliferation in HNSCC cells dose- and time- dependently.

### Nisin-mediated calcium influxes and apoptosis are blocked by a calcium channel blocker

**Figure 2:**
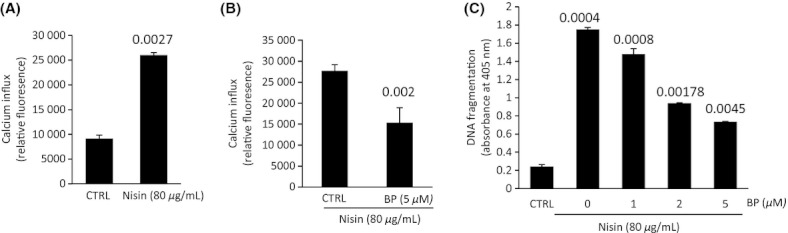
Nisin-mediated calcium influxes and apoptosis are blocked by bepridil (BP), a calcium channel blocker. (A) and (B) Calcium influx and (C) DNA fragmentation levels in UM-SCC-17B cells after treatment with nisin (80 μg/mL) and bepridil as indicated for 24 h. *P* values for each data set are indicated individually.

Nisin is known to alter the influx of ions through its effects on membrane phospholipid reorganization [24]. To determine whether nisin's ability to induce apoptosis in HNSCC cells was dependent on nisin's ability to alter calcium influxes in these cells, calcium influx levels were measured following nisin treatment. Nisin treatment significantly increased calcium influxes in HNSCC cells, and treatment with a calcium channel blocker, Bepridil, blocked the nisin-mediated calcium influx (Fig. 2). Bepridil also blocked the nisin-mediated DNA fragmentation or apoptosis in HNSCC cells dose- dependently. These data indicate that nisin mediates apoptosis in HNSCC cells via changes in calcium influxes.

### Nisin reduces HNSCC cell proliferation by arresting cells in the G2 phase of the cell cycle

**Figure 3:**
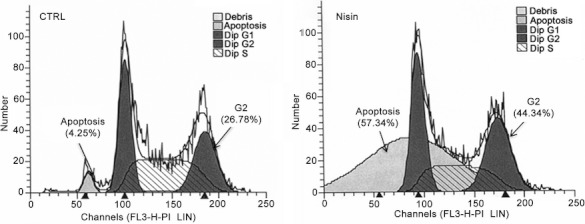
Nisin induces cell cycle arrest. Cell cycle analysis of UM-SCC-17B cells after treatment with nisin (80 μg/mL) or control for 24 h.

To further explore nisin's effects on HNSCC cell proliferation, cell cycle status was examined (Fig. 3). Treatment of HNSCC cells with nisin induced cell cycle arrest in the G2 phase with concomitant decreases in Cdc2 phosphorylation, a cell cycle checkpoint marker (Figs. 3 and S2). In addition, in agreement with the DNA fragmentation data (Fig. 1), nisin concomitantly increased levels/cleavage of the apoptotic markers cPARP and active caspase-3 (Fig. S2).

### CHAC1, a cation transport regulator, is upregulated by nisin treatment

**Figure 4:**
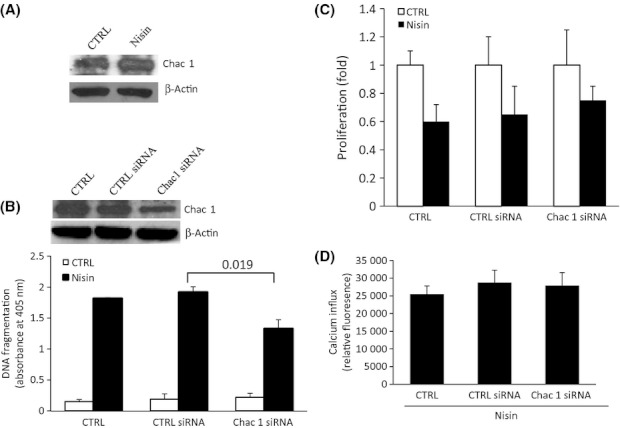
CHAC1 suppression inhibits nisin-induced DNA fragmentation. (A) Immunoblot showing CHAC1 expression in UM-SCC-17B cells after treatment with nisin (80 μg/mL) for 24 h. β-Actin served as loading control. (B) DNA fragmentation, (C) fold change in proliferation, and (D) calcium influx levels in UM-SCC-17B cells after transfection with CTRL siRNA or CHAC1 siRNA and treated with nisin (80 μg/mL) for 24 h. Inset, immunoblot showing CHAC1 levels after transfection with CHAC1 siRNA. *P* values for each data set are indicated individually.

To examine the mechanism by which nisin mediates its proapoptotic and antiproliferative effects on HNSCC cells, gene expression arrays were used to explore potential genes altered by nisin treatment in these cells. Using Affymetrix gene arrays that examine over 39,000 genes, *CHAC1*, a cation transport regulator and apoptosis mediator [17] was identified as the most highly upregulated (fourfold) gene in nisin-treated HNSCC cells compared with untreated controls (Table 1). CHAC1 protein levels were also increased in these cells upon nisin treatment as confirmed by Western blotting (Fig. 4). Other genes that were also upregulated or downregulated by nisin treatment up to twofold were included in Table 1. Nisin-altered genes included those in apoptotic and cell cycle pathways, membrane physiology, ion transport, energy and nutrient pathways, and protein binding and signal transduction pathways.

**Table 1:** Gene microarray table illustrating fold change in gene expression of HNSCC cells treated with nisin compared with untreated controls

Symbol	Description	Gene ontology	Fold change
CHAC1	ChaC, cation transport regulator homolog 1 (*Escherichia coli*)	Protein binding	4.42976
PDE4C	Phosphodiesterase 4C, cAMP specific (phosphodiesterase E1 dunce homolog, Drosophila)	Signal transduction	4.29073
LGALS1	Lectin, galactoside-binding, soluble, 1 (galectin 1)	Sugar binding	3.64717
		Regulation of apoptosis	
S100P	S100 calcium-binding protein P	Calcium ion binding	3.21482
		Calcium-dependent protein binding	
STC2	Stanniocalcin 2	Cell surface receptor linked signal transduction	3.17316
		Response to nutrient	
PILRA	Paired immunoglobulin-like type 2 receptor α	Protein binding	2.58501
		Signal transduction	
		Integral to membrane	
ARC	Activity-regulated cytoskeleton-associated protein	Membrane	2.35159
		Cell junction	
PYGM	Phosphorylase, glycogen; muscle (McArdle syndrome, glycogen storage disease type V)	Carbohydrate metabolic process	2.34776
		Glycogen catabolic process	
		Glycogen phosphorylase activity	
RAB3A	RAB3A, member RAS oncogene family	Membrane	2.26615
TEX14	Testis expressed 14	ATP binding	2.24405
SDPR	Serum deprivation response (phosphatidylserine-binding protein)	Phosphatidylserine binding	2.20425
		Membrane fraction	
PLA2G12B	Phospholipase A2, group XIIB	Phospholipase A2 activity	2.18512
		Calcium ion binding	
		Lipid catabolic process	
ITGAM	Integrin, alpha M (complement component 3 receptor 3 subunit)	Magnesium ion binding	2.13638
		Calcium ion binding	
		External side of plasma membrane	
		Integral to membrane	
SESN2	Sestrin 2	Cell cycle arrest	2.13004
PRR3	Proline rich 3	Zinc ion binding	2.09132
		Metal ion binding	
CYP2B6	Cytochrome P450, family 2, subfamily B, polypeptide 6	Endoplasmic reticulum	2.06563
		Electron transport	
		Membrane	
		Metal ion binding	
LAMA4	Laminin, alpha 4	Receptor binding	2.03249
NR4A2	Nuclear receptor subfamily 4, group A, member 2	Steroid hormone receptor activity	2.02835
		Zinc ion binding	
		Metal ion binding	
ZNF34	Zinc finger protein 34	Zinc ion binding	2.02091
		Metal ion binding	
TNFSF11	Tumor necrosis factor (ligand) superfamily, member 11	Integral to plasma membrane	2.00225
		Membrane	
		Cell differentiation	
ZBTB24	Zinc finger and BTB domain containing 24	Metal ion binding	−3.76108
ZBTB24	Zinc finger and BTB domain containing 24	Metal ion binding	−3.5799
ARHGAP11B	Rho GTPase activating protein 11B	Signal transduction	−2.5269
ALS2	Amyotrophic lateral sclerosis 2 (juvenile)	Response to oxidative stress	−2.52641
TRSPAP1	tRNA selenocysteine-associated protein 1	Protein binding	2.43763
MARS2	Methionyl-tRNA synthetase 2, mitochondrial	Mitochondrion	−2.36375
		Mitochondrial matrix	
FST	Follistatin	Negative regulation of cell differentiation	−2.34739
SETX	Senataxin	ATP binding	−2.30816
		Cell death	
PARG	Poly (ADP-ribose) glycohydrolase	Response to DNA damage stimulus	−2.27345
KCTD1	Potassium channel tetramerization domain containing 1	Voltage-gated potassium channel activity	−2.26108
		Protein binding	
		Potassium ion transport	
		Voltage-gated potassium channel complex	
		Membrane	
PPAN	Peter pan homolog (Drosophila)	Protein binding	−2.2312
ZNF718	Zinc finger protein 718	Zinc ion binding	−2.1316
		Metal ion binding	
MED13	Mediator complex subunit 13	Receptor activity	−2.11608
		Transcription	
CCNT2	Cyclin T2	Cell cycle	−2.01302
		Cell division	

### Nisin-mediated effects on cell apoptosis are blocked by suppressing CHAC1

To functionally determine whether nisin effects on HNSCC cell apoptosis and proliferation were mediated by CHAC1, CHAC1 levels were suppressed in the context of nisin treatment. CHAC1 suppression with siRNA reversed the nisin-mediated effects on cell apoptosis, and therefore decreased HNSCC cell apoptosis upon nisin treatment compared with controls (Fig. 4). However, CHAC1 suppression did not affect the nisin-mediated effects on HNSCC cell proliferation and calcium influx levels. Thus, CHAC1, in part, mediates the nisin effects on HNSCC cell apoptosis, and these CHAC1-mediated effects are independent of calcium influxes.

### Nisin-mediated effects on cell apoptosis are enhanced by CHAC1 overexpression

**Figure 5:**
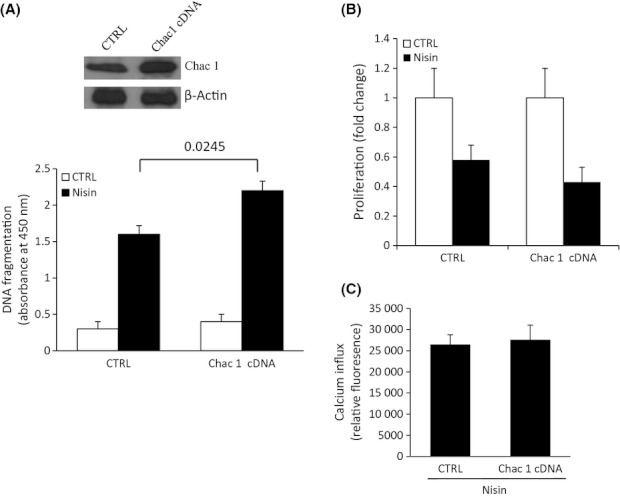
CHAC1 overexpression increases nisin-induced DNA fragmentation. (A) DNA fragmentation, (B) fold change in proliferation, and (C) calcium influx levels in UM-SCC-17B cells after transfection with CHAC1 cDNA and treated with nisin (80 μg/mL) for 24 h. Inset, immunoblot showing CHAC1 levels after transfection with CHAC1 cDNA. *P* values for each data set are indicated individually.

To further confirm that nisin-mediated effects on HNSCC cell apoptosis are mediated by CHAC1, CHAC1 overexpression was examined in the context of nisin treatment (Fig. 5). CHAC1 overexpression enhanced nisin's effects on cell apoptosis without affecting cell proliferation and calcium influx levels. Taken in aggregate, these data indicate that nisin mediates its effects on HNSCC cell apoptosis via CHAC1, and CHAC1 functions independent of calcium influxes.

### Nisin reduces HNSCC tumor burden in vivo

Given nisin's in vitro effects on enhancing apoptosis and blocking cell proliferation in HNSCC cells, nisin's in vivo effects were next examined using a floor-of-mouth oral cancer xenograft mouse model. Given that nisin doses of approximately 80 mg/kg did not exhibit adverse effects in rats [21], we tested a 150 mg/kg dose of nisin administered over the course of 3 weeks and did not observe adverse effects in mice, which exhibited normal weight gain and normal organ histology (Fig. S1A).

**Figure 6:**
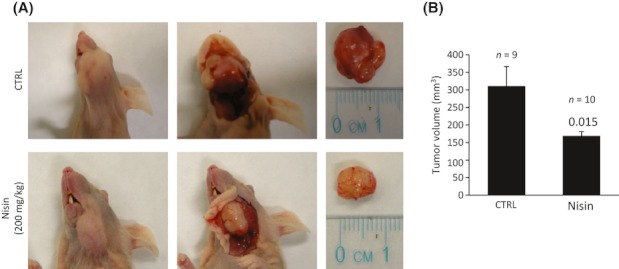
Nisin administration reduces HNSCC tumor burden in mice. (A) Mice were injected with UM-SCC-17B cells then administered either water (CTRL) or nisin (200 mg/kg per day) for 3 weeks. Left panels show superficial tumors, middle panels show dissected tumors, and right panels show dissected and isolated tumors. (B) Tumor volumes for mice injected with UM-SCC-17B cells and administered either water (CTRL) or nisin (200 mg/kg per day) for 3 weeks. *P* values for each data set are indicated individually.

In initial experiments, tumors were first established after approximately 3 weeks following tumor cell inoculation, then tumor-bearing mice were equally distributed into two groups and nisin or water/control was administered to mice via daily oral gavage for 3 weeks. Mice that received nisin treatment exhibited statistically significant reduced tumor volumes compared with controls (Fig. 6). In the next set of experiments, where mice were treated with nisin or water/control by oral gavage for 3 weeks prior to and 3 weeks after tumor cell inoculation, nisin pretreatment again resulted in statistically significant reduction in tumor volumes (Fig. S1B).

### CHAC1 stable suppression reduces tumor burden in vivo

**Figure 7:**
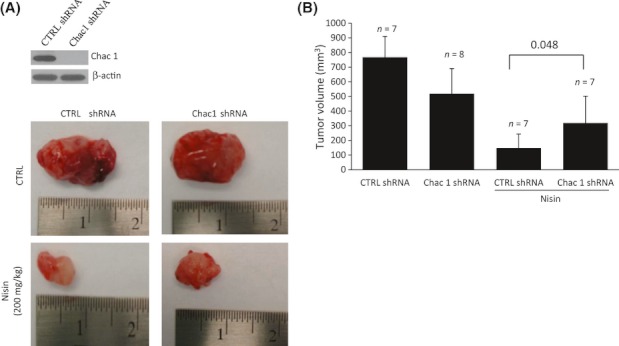
CHAC1 downregulation counteract the tumor-suppressive effects of nisin in vivo. (A) Top, immunoblots show CHAC1 levels after stable CHAC1 suppression using lentiviral particles (CTRL shRNA or CHAC1-shRNA) in UM-SCC-17B cells. Bottom, images show the dissected tumors of mice injected with CTRL shRNA or CHAC1-shRNA and administered either water (CTRL) or nisin (200 mg/kg per day) for 3 weeks. (B) Tumor volumes for mice injected with CTRL shRNA or CHAC1-shRNA and administered water (CTRL) or nisin (200 mg/kg per day) for 3 weeks. *P* values for each data set are indicated individually.

As in the in vitro data, CHAC1 suppression was next examined in the in vivo floor-of-the-mouth mouse model. Mice inoculated with HNSCC cells exhibiting stable CHAC1 suppression and treated with nisin exhibited statistically significant increased tumor volumes compared with controls, indicating that nisin effects are mediated via CHAC1 in vivo (Fig. 7). CHAC1 suppression alone had no statistically significant effects on tumor volumes. In agreement with other in vivo data, nisin treatment again reduced overall tumor burden compared with the nonnisin-treated groups.

## Discussion

These data demonstrate that nisin induces preferential apoptosis and decreased cell proliferation in HNSCC cells compared with primary keratinocytes due to increases in intracellular calcium, induction of cell cycle arrest, and activation of CHAC1 in HNSCC cells. Furthermore, nisin similarly prevents and inhibits the growth of HNSCC tumors in vivo via induction of CHAC1 expression, as its suppression significantly increases tumor volume. CHAC1 is a known cation transport regulator and apoptosis mediator; however, its effects on cancer cell apoptosis have never been examined. Our studies are, therefore, the first to report this important new role for CHAC1 in this process. Future studies will help determine the potential use of CHAC1 as an adjunctive therapeutic target for HNSCC.

Calcium influx inhibitors have been known to alter tumorigenic properties. Nifedipine, a calcium channel blocker, that inhibits the influx of intracellular calcium ions within vascular smooth muscle and protects against angina and hypertension, reduces HNSCC cell migration [25]. In a recent Phase I clinical trial, a novel calcium influx inhibitor, carboxyamidotriazole, was shown to stabilize tumor progression in approximately 50% of patients with advanced disease with low toxicity, indicating the potential use of calcium influx inhibitors in managing advanced-stage cancers [26]. Carboxyamidotriazole can also inhibit the proliferation, migration, and invasive potential of HNSCC cells in vitro plus their production of MMP-2 and MMP-2 [27]. Maslinic acid, a triterpenoid found in high concentration in olives, induces calcium influxes that lead to apoptosis of salivary gland adenoid cystic carcinoma [28]. Thus, the current findings are in agreement with these and other studies demonstrating that increased calcium influxes regulate several tumor cell properties that can be abrogated with the use of calcium influx inhibitors.

The mechanistic basis of nisin's selectivity for HNSCC cells versus primary keratinocytes may stem from the known structural differences in plasma membrane composition between these two cell types. Specifically, the phospholipid/lipoprotein content and composition of HNSCC/OSCC cells is known to be functionally different from that of primary keratinocytes [10–15]. Furthermore, in preliminary Affymetrix gene array data, nisin treatment of HNSCC cells showed a preponderance of altered genes that regulate calcium/ions and lipid/membrane function. The most highly upregulated gene was CHAC1, a cation transport regulator that promotes apoptosis and is activated by oxidized phospholipids. As nisin is known to preferentially interact with and alter membrane phospholipids, especially phosphatidylcholine, it is possible that CHAC1 is a downstream nisin target that responds to alterations in membrane phospholipids. It remains to be determined what the contribution of phospholipid composition is to nisin-mediated effects in this process.

This is the first time that an antibacterial food preservative agent has been shown to effectively reduce and prevent tumorigenic properties in vitro and in vivo. As nisin is already safely consumed by humans and is approved by the FDA and WHO for human consumption, nisin lends itself to a natural transition as an OSCC therapeutic. Determining the optimal therapeutic dose for treatment of human OSCC is the next critical step in its validation process.

## Supporting Information

**Figure S1.** Nisin treatment does not alter liver and kidney histological morphology and preloading with nisin reduces tumor volume. (A) Representative hematoxylin and eosin staining of liver and kidney tissue sections from mice administered 150 mg/kg per day of nisin for 3 weeks (*n* = 3 mice). (B) Tumor volumes for mice administered water (CTRL) or nisin (200 mg/kg per day) for 3 weeks pre- and post injection of UM-SCC-17B cells. *P* values for each data set are indicated individually.

**Figure S2.** Nisin inhibits Cdc2 phosphorylation but promotes PARP and caspase-3 cleavage. Immunoblots showing (A) Cdc2 and p-Cdc2 and (B) active/cleaved PARP and caspase-3 expression in control (CTRL) and nisin-treated UM-SCC-17B cells. β-Actin served as a loading control.
