# Supplementary material for: Nisin, an apoptogenic bacteriocin and food preservative, attenuates HNSCC tumorigenesis via CHAC1
Source: Cancer Med. 2012 Oct 2;1(3):295–305. doi: 10.1002/cam4.35 (PMC3544465; doi:10.1002/cam4.35)
Supplement: Supplementary file 1 [file cam40001-0295-SD1.pptx]

## Slide 1
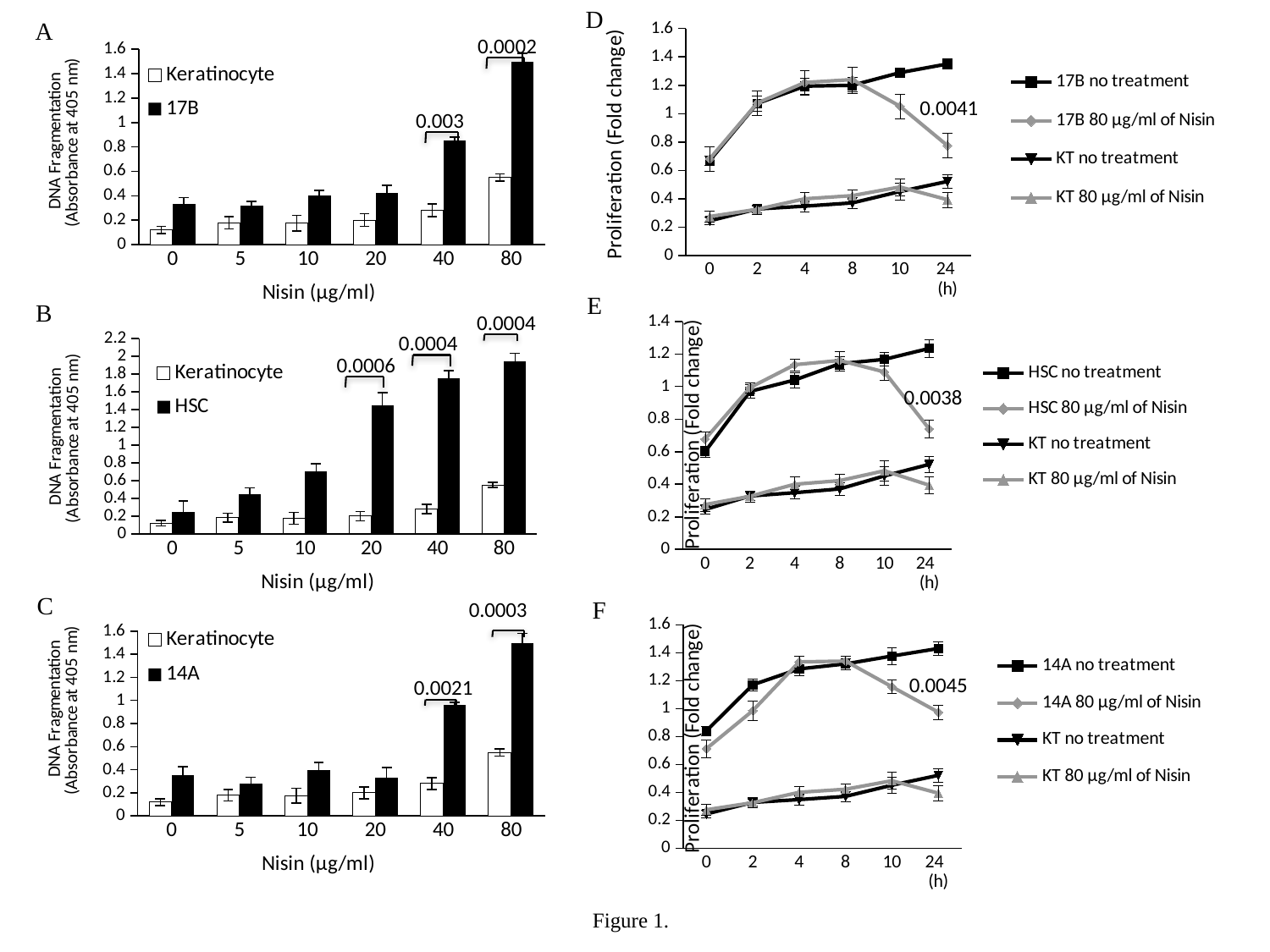

D
A
### Chart
| Category | 17B no treatment | 17B 80 µg/ml of Nisin | KT no treatment | KT 80 µg/ml of Nisin |
|---|---|---|---|---|
| 0 | 0.670000000000001 | 0.682 | 0.245 | 0.275 |
| 2 | 1.072 | 1.075 | 0.328000000000001 | 0.326000000000001 |
| 4 | 1.194000000000002 | 1.22 | 0.348 | 0.4 |
| 8 | 1.2 | 1.24 | 0.371 | 0.422 |
| 10 | 1.288999999999997 | 1.052999999999997 | 0.451 | 0.483 |
| 24 (h) | 1.35 | 0.775000000000001 | 0.522 | 0.394000000000001 |0.0002
### Chart
| Category | Keratinocyte | 17B |
|---|---|---|
| 0.0 | 0.12 | 0.330000000000004 |
| 5.0 | 0.18 | 0.320000000000004 |
| 10.0 | 0.175 | 0.4 |
| 20.0 | 0.2 | 0.42 |
| 40.0 | 0.28 | 0.850000000000002 |
| 80.0 | 0.55 | 1.5 |0.003
E
B
0.0004
### Chart
| Category | HSC no treatment | HSC 80 µg/ml of Nisin | KT no treatment | KT 80 µg/ml of Nisin |
|---|---|---|---|---|
| 0 | 0.600000000000001 | 0.677000000000001 | 0.245 | 0.275 |
| 2 | 0.97 | 0.993 | 0.328000000000001 | 0.326000000000001 |
| 4 | 1.04 | 1.134 | 0.348 | 0.4 |
| 8 | 1.14 | 1.16 | 0.371 | 0.422 |
| 10 | 1.167 | 1.09 | 0.451 | 0.483 |
| 24 (h) | 1.234 | 0.740000000000001 | 0.522 | 0.394000000000001 |0.0004
### Chart
| Category | Keratinocyte | HSC |
|---|---|---|
| 0.0 | 0.12 | 0.25 |
| 5.0 | 0.18 | 0.45 |
| 10.0 | 0.175 | 0.700000000000002 |
| 20.0 | 0.2 | 1.45 |
| 40.0 | 0.28 | 1.75 |
| 80.0 | 0.55 | 1.940000000000012 |0.0006
C
F
### Chart
| Category | Keratinocyte | 14A |
|---|---|---|
| 0.0 | 0.12 | 0.35 |
| 5.0 | 0.18 | 0.28 |
| 10.0 | 0.175 | 0.4 |
| 20.0 | 0.2 | 0.330000000000004 |
| 40.0 | 0.28 | 0.960000000000002 |
| 80.0 | 0.55 | 1.5 |
### Chart
| Category | 14A no treatment | 14A 80 µg/ml of Nisin | KT no treatment | KT 80 µg/ml of Nisin |
|---|---|---|---|---|
| 0 | 0.840000000000001 | 0.712000000000001 | 0.245 | 0.275 |
| 2 | 1.170000000000002 | 0.985 | 0.328000000000001 | 0.326000000000001 |
| 4 | 1.284 | 1.334 | 0.348 | 0.4 |
| 8 | 1.32 | 1.34 | 0.371 | 0.422 |
| 10 | 1.376 | 1.156 | 0.451 | 0.483 |
| 24 (h) | 1.43 | 0.974000000000001 | 0.522 | 0.394000000000001 |0.0021
Figure 1.

## Slide 2
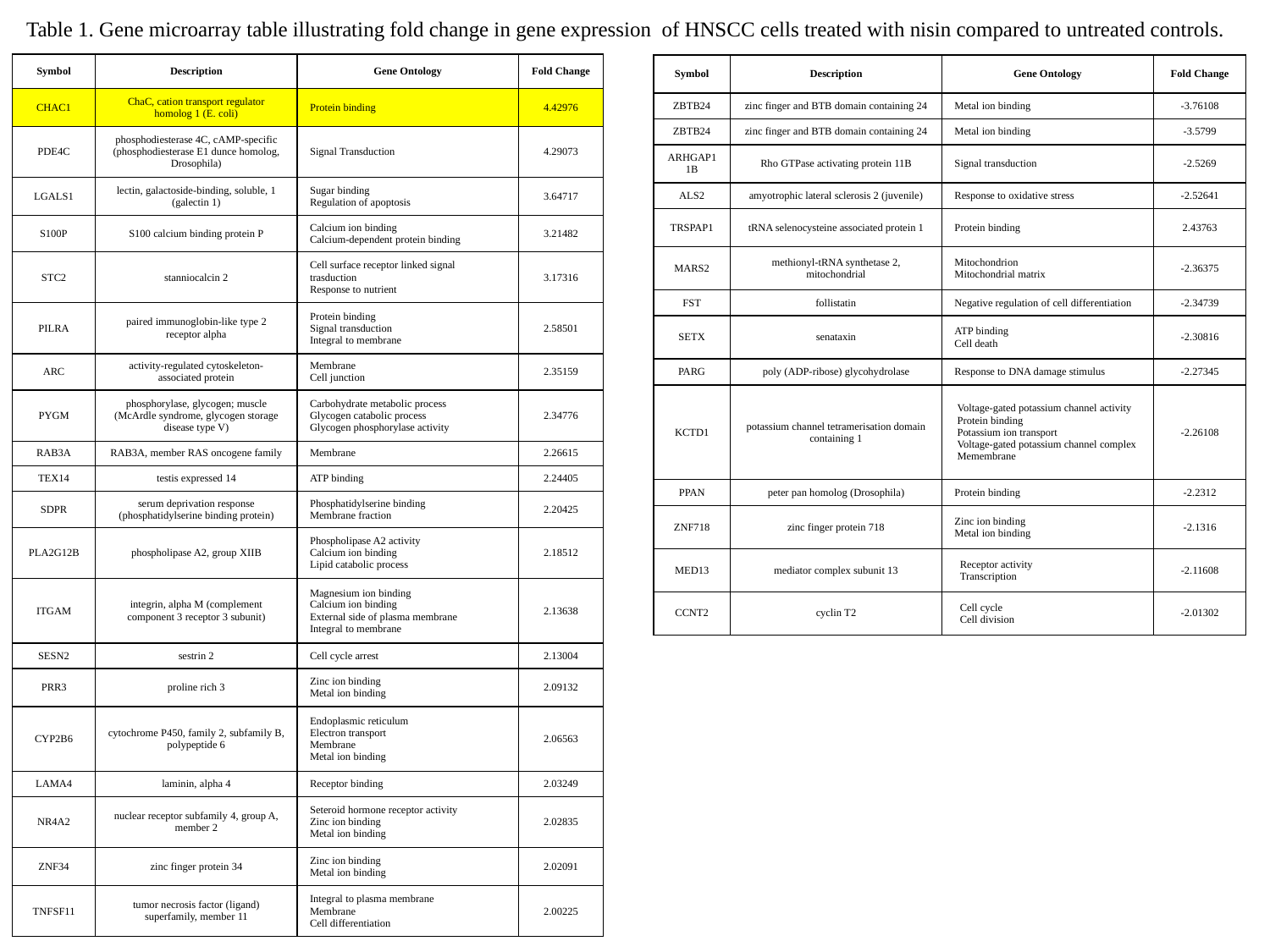

Table 1. Gene microarray table illustrating fold change in gene expression of HNSCC cells treated with nisin compared to untreated controls.
| Symbol | Description | Gene Ontology | Fold Change |
| --- | --- | --- | --- |
| CHAC1 | ChaC, cation transport regulator homolog 1 (E. coli) | Protein binding | 4.42976 |
| PDE4C | phosphodiesterase 4C, cAMP-specific (phosphodiesterase E1 dunce homolog, Drosophila) | Signal Transduction | 4.29073 |
| LGALS1 | lectin, galactoside-binding, soluble, 1 (galectin 1) | Sugar binding Regulation of apoptosis | 3.64717 |
| S100P | S100 calcium binding protein P | Calcium ion binding Calcium-dependent protein binding | 3.21482 |
| STC2 | stanniocalcin 2 | Cell surface receptor linked signal trasduction Response to nutrient | 3.17316 |
| PILRA | paired immunoglobin-like type 2 receptor alpha | Protein binding Signal transduction Integral to membrane | 2.58501 |
| ARC | activity-regulated cytoskeleton-associated protein | Membrane Cell junction | 2.35159 |
| PYGM | phosphorylase, glycogen; muscle (McArdle syndrome, glycogen storage disease type V) | Carbohydrate metabolic process Glycogen catabolic process Glycogen phosphorylase activity | 2.34776 |
| RAB3A | RAB3A, member RAS oncogene family | Membrane | 2.26615 |
| TEX14 | testis expressed 14 | ATP binding | 2.24405 |
| SDPR | serum deprivation response (phosphatidylserine binding protein) | Phosphatidylserine binding Membrane fraction | 2.20425 |
| PLA2G12B | phospholipase A2, group XIIB | Phospholipase A2 activity Calcium ion binding Lipid catabolic process | 2.18512 |
| ITGAM | integrin, alpha M (complement component 3 receptor 3 subunit) | Magnesium ion binding Calcium ion binding External side of plasma membrane Integral to membrane | 2.13638 |
| SESN2 | sestrin 2 | Cell cycle arrest | 2.13004 |
| PRR3 | proline rich 3 | Zinc ion binding Metal ion binding | 2.09132 |
| CYP2B6 | cytochrome P450, family 2, subfamily B, polypeptide 6 | Endoplasmic reticulum Electron transport Membrane Metal ion binding | 2.06563 |
| LAMA4 | laminin, alpha 4 | Receptor binding | 2.03249 |
| NR4A2 | nuclear receptor subfamily 4, group A, member 2 | Seteroid hormone receptor activity Zinc ion binding Metal ion binding | 2.02835 |
| ZNF34 | zinc finger protein 34 | Zinc ion binding Metal ion binding | 2.02091 |
| TNFSF11 | tumor necrosis factor (ligand) superfamily, member 11 | Integral to plasma membrane Membrane Cell differentiation | 2.00225 |
| Symbol | Description | Gene Ontology | Fold Change |
| --- | --- | --- | --- |
| ZBTB24 | zinc finger and BTB domain containing 24 | Metal ion binding | -3.76108 |
| ZBTB24 | zinc finger and BTB domain containing 24 | Metal ion binding | -3.5799 |
| ARHGAP11B | Rho GTPase activating protein 11B | Signal transduction | -2.5269 |
| ALS2 | amyotrophic lateral sclerosis 2 (juvenile) | Response to oxidative stress | -2.52641 |
| TRSPAP1 | tRNA selenocysteine associated protein 1 | Protein binding | 2.43763 |
| MARS2 | methionyl-tRNA synthetase 2, mitochondrial | Mitochondrion Mitochondrial matrix | -2.36375 |
| FST | follistatin | Negative regulation of cell differentiation | -2.34739 |
| SETX | senataxin | ATP binding Cell death | -2.30816 |
| PARG | poly (ADP-ribose) glycohydrolase | Response to DNA damage stimulus | -2.27345 |
| KCTD1 | potassium channel tetramerisation domain containing 1 | Voltage-gated potassium channel activity Protein binding Potassium ion transport Voltage-gated potassium channel complex Memembrane | -2.26108 |
| PPAN | peter pan homolog (Drosophila) | Protein binding | -2.2312 |
| ZNF718 | zinc finger protein 718 | Zinc ion binding Metal ion binding | -2.1316 |
| MED13 | mediator complex subunit 13 | Receptor activity Transcription | -2.11608 |
| CCNT2 | cyclin T2 | Cell cycle Cell division | -2.01302 |

## Slide 3
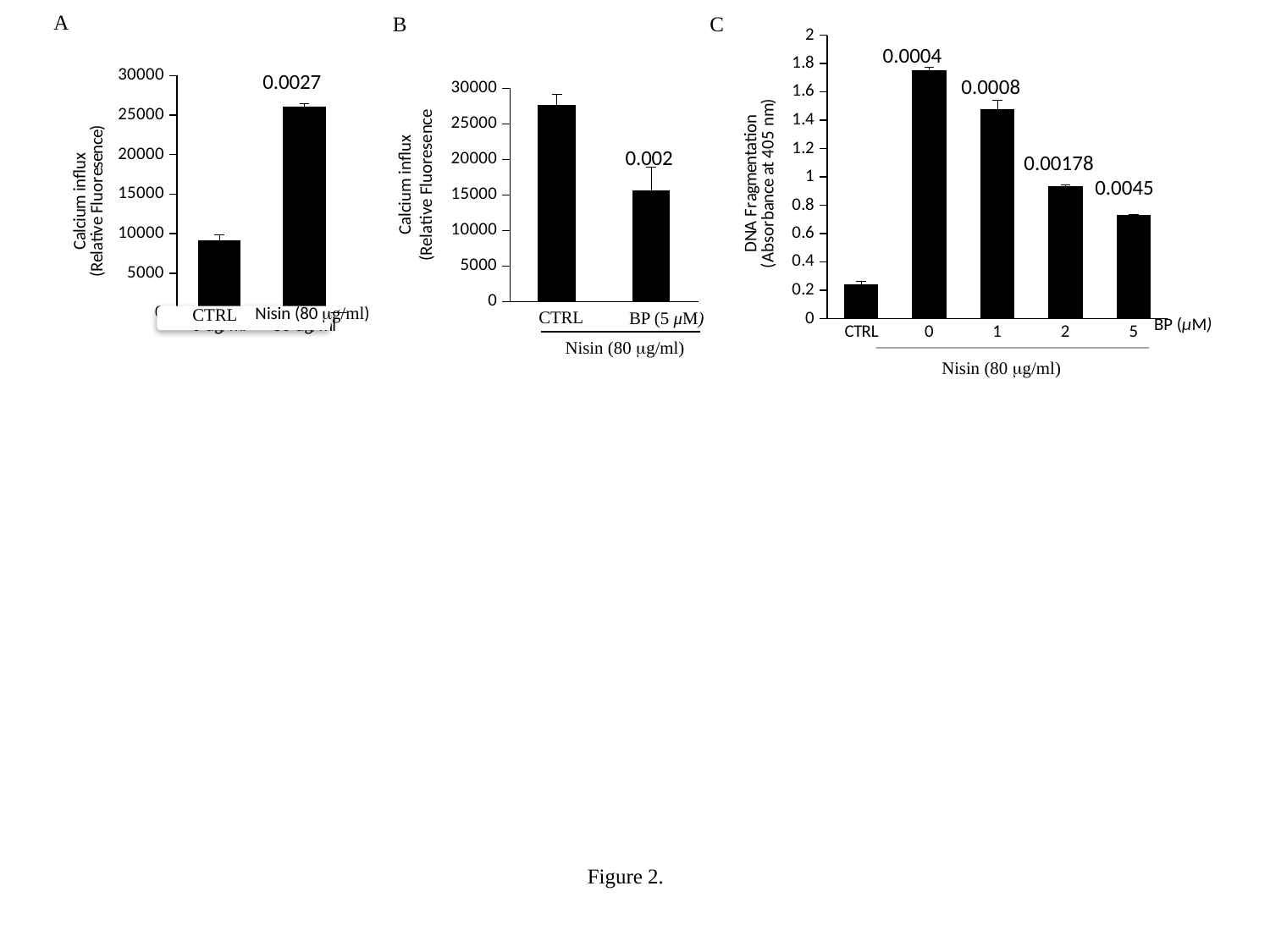

A
B
C
### Chart
| Category | |
|---|---|
| CTRL | 0.243 |
| 0 | 1.75 |
| 1 | 1.478 |
| 2 | 0.937000000000001 |
| 5 | 0.733000000000001 |0.0004
0.0008
0.00178
0.0045
BP (μM)
### Chart
| Category | |
|---|---|
| 0 ug/ml | 9148.33 |
| 80 ug/ml | 26020.0 |
### Chart
| Category | |
|---|---|
| No treat. | 27640.0 |
| 5  μM Bepridil | 15590.0 |Calcium influx
(Relative Fluoresence
Nisin (80 mg/ml)
CTRL
CTRL
BP (5 μM)
Nisin (80 mg/ml)
Nisin (80 mg/ml)
Figure 2.

## Slide 4
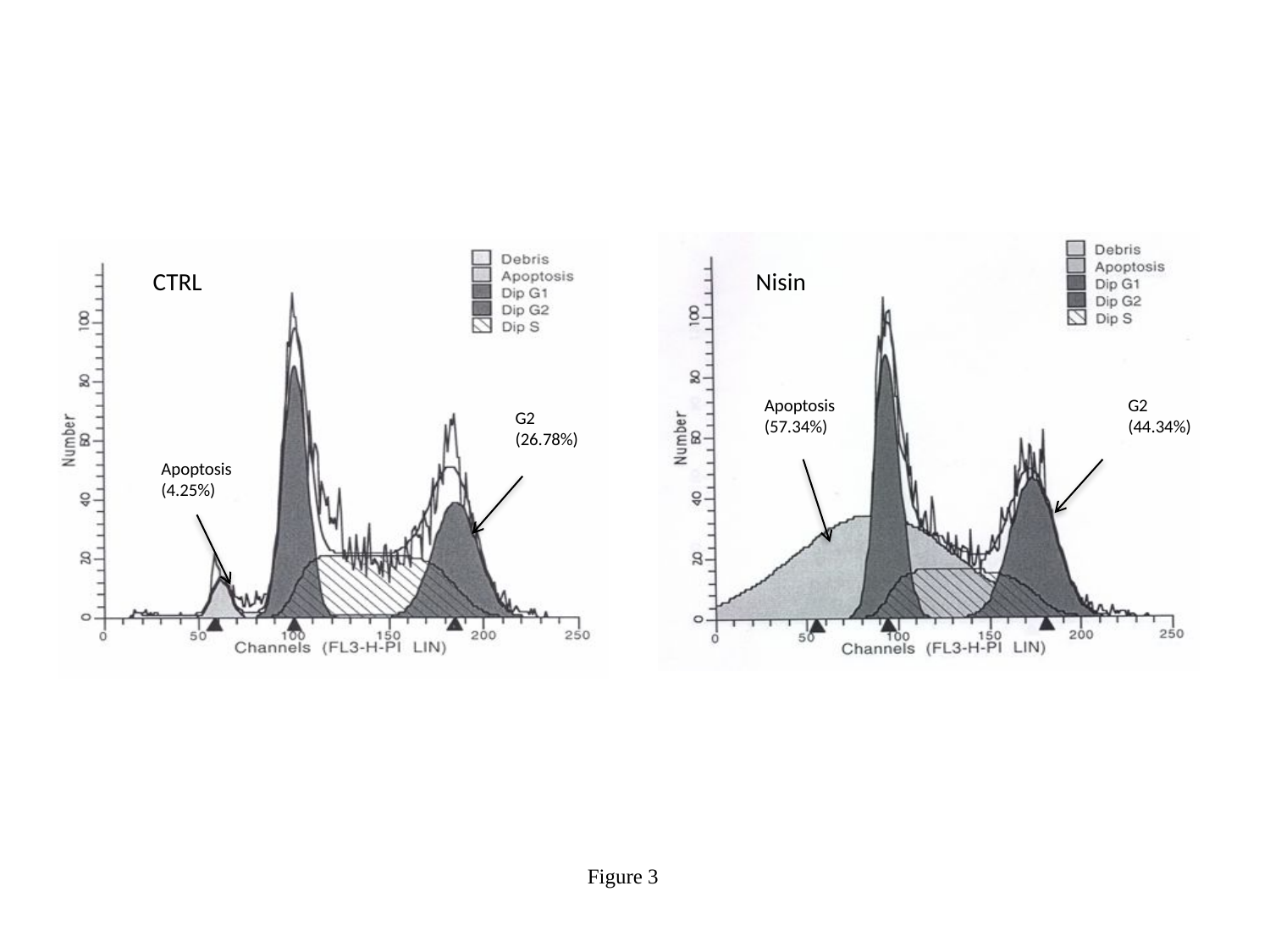

Nisin
Apoptosis
(57.34%)
G2
(44.34%)
CTRL
G2
(26.78%)
Apoptosis
(4.25%)
Figure 3

## Slide 5
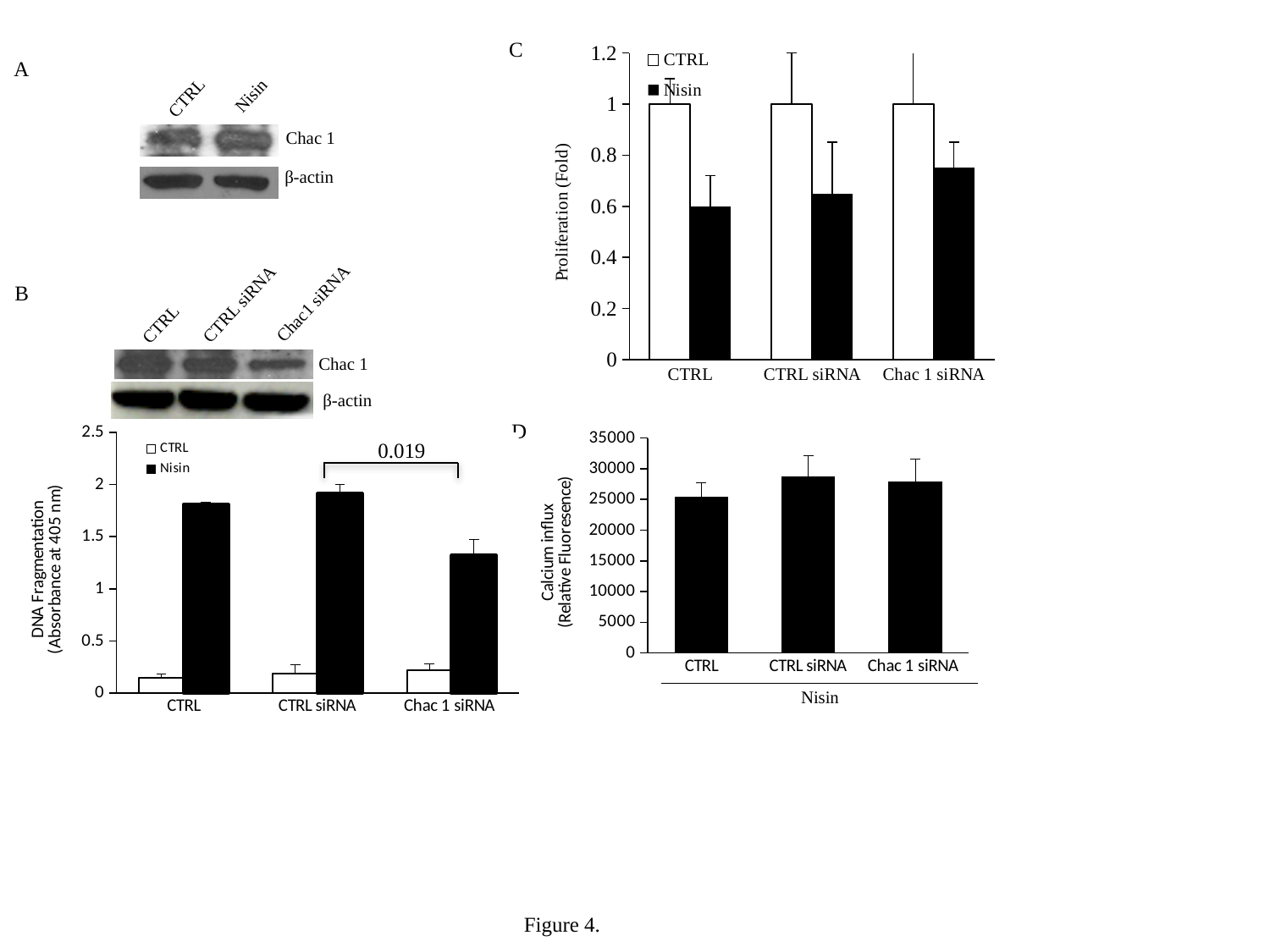

C
### Chart
| Category | CTRL | Nisin |
|---|---|---|
| CTRL | 1.0 | 0.600000000000001 |
| CTRL siRNA | 1.0 | 0.650000000000001 |
| Chac 1 siRNA | 1.0 | 0.750000000000001 |A
Nisin
CTRL
Chac 1
β-actin
CTRL siRNA
Chac1 siRNA
CTRL
Chac 1
B
β-actin
D
### Chart
| Category | CTRL | Nisin |
|---|---|---|
| CTRL | 0.15 | 1.808999999999997 |
| CTRL siRNA | 0.19 | 1.911 |
| Chac 1 siRNA | 0.22 | 1.32 |0.019
### Chart
| Category | |
|---|---|
| CTRL | 25463.0 |
| CTRL siRNA | 28766.0 |
| Chac 1 siRNA | 27892.0 |Nisin
Figure 4.

## Slide 6
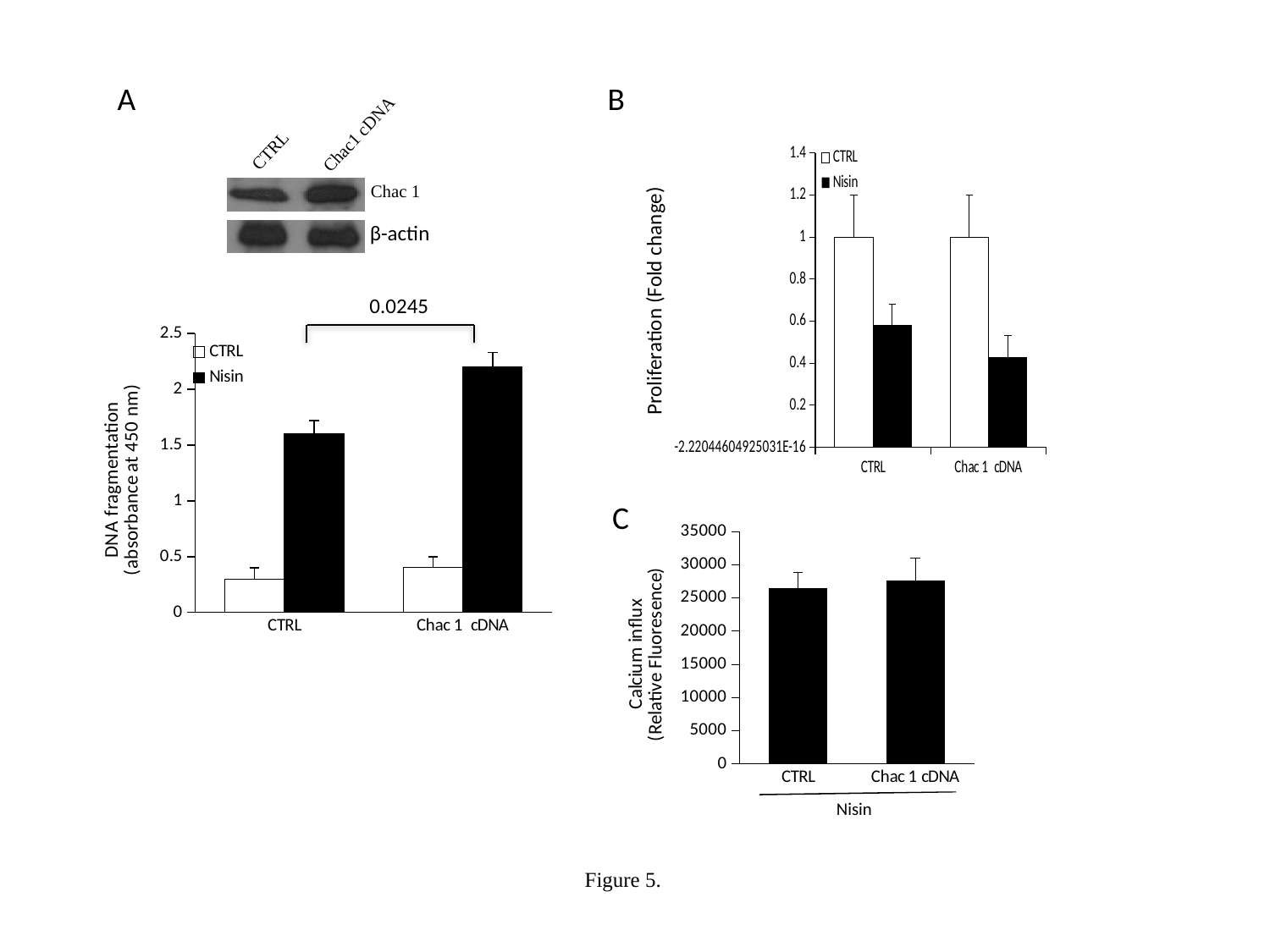

A
B
Chac1 cDNA
CTRL
### Chart
| Category | CTRL | Nisin |
|---|---|---|
| CTRL | 1.0 | 0.58 |
| Chac 1 cDNA | 1.0 | 0.43 |Chac 1
β-actin
Proliferation (Fold change)
0.0245
### Chart
| Category | CTRL | Nisin |
|---|---|---|
| CTRL | 0.3 | 1.6 |
| Chac 1 cDNA | 0.4 | 2.2 |C
D
### Chart
| Category | |
|---|---|
| CTRL | 26463.0 |
| Chac 1 cDNA | 27589.0 |Nisin
Figure 5.

## Slide 7
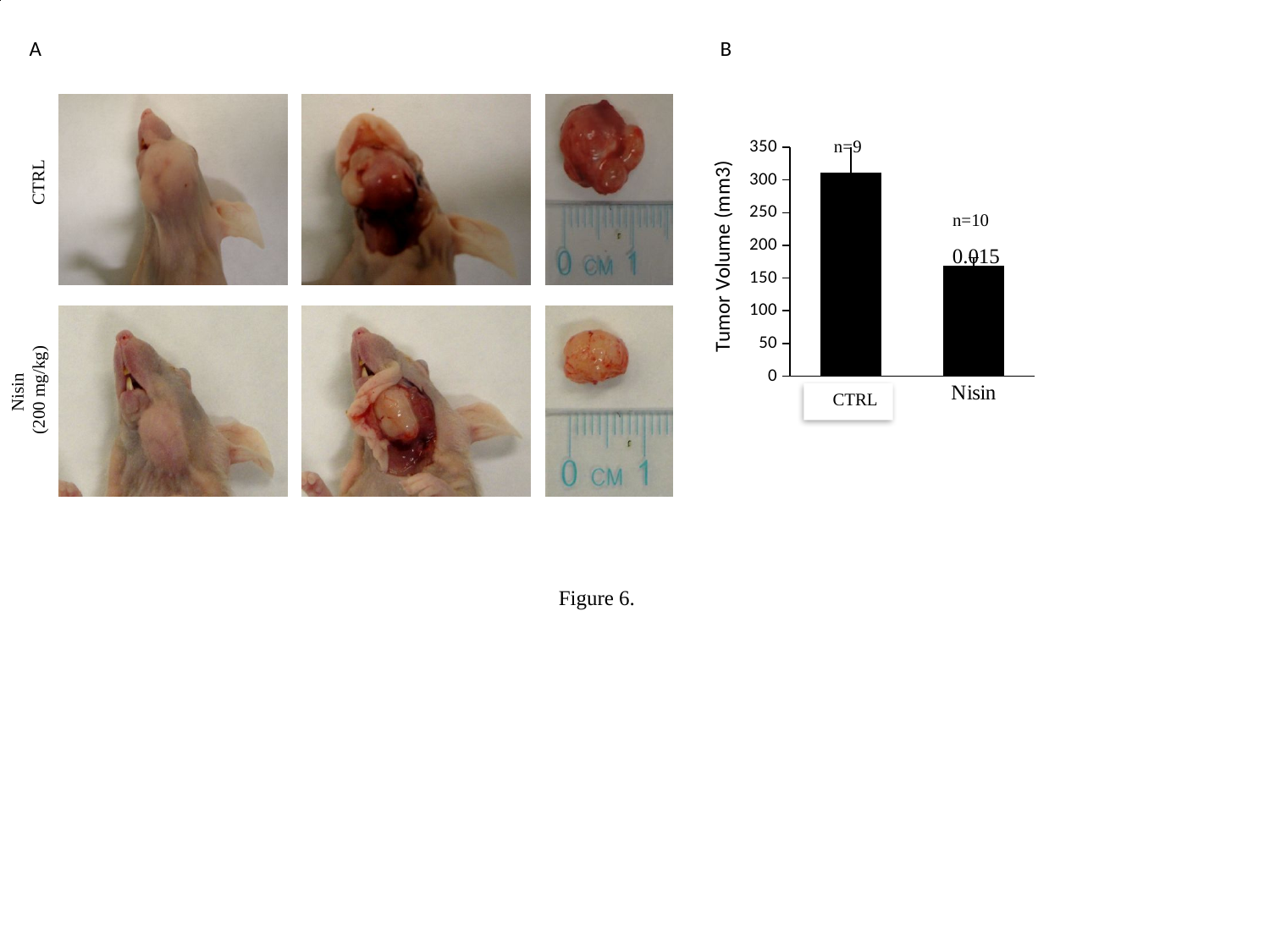

### Chart
| Category | |
|---|---|
| Control | 311.02 |
| Nisin | 168.91 |A
B
n=9
### Chart
| Category | |
|---|---|
| Control | 311.02 |
| Nisin | 168.91 |n=10
0.015
CTRL
Nisin
(200 mg/kg)
CTRL
Figure 6.

## Slide 8
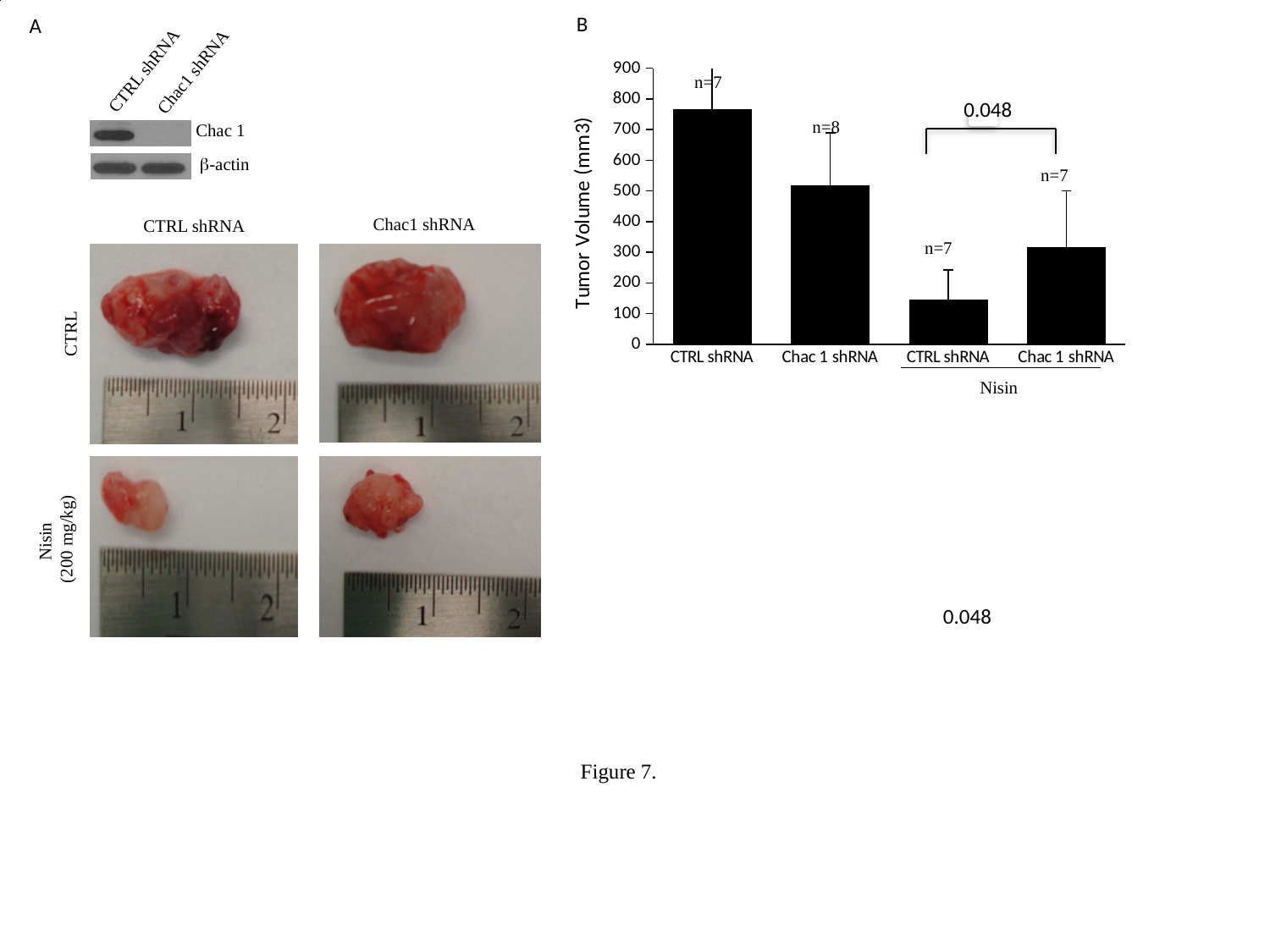

### Chart
| Category | |
|---|---|
| Control | 311.02 |
| Nisin | 168.91 |B
A
CTRL shRNA
Chac1 shRNA
Chac 1
b-actin
### Chart
| Category | | |
|---|---|---|
| CTRL shRNA | None | 767.0 |
| Chac 1 shRNA | None | 518.0 |
| CTRL shRNA | None | 147.0 |
| Chac 1 shRNA | None | 318.0 |n=7
0.048
*
n=8
n=7
Chac1 shRNA
CTRL shRNA
n=7
CTRL
Nisin
Nisin
(200 mg/kg)
0.048
Figure 7.

## Slide 9
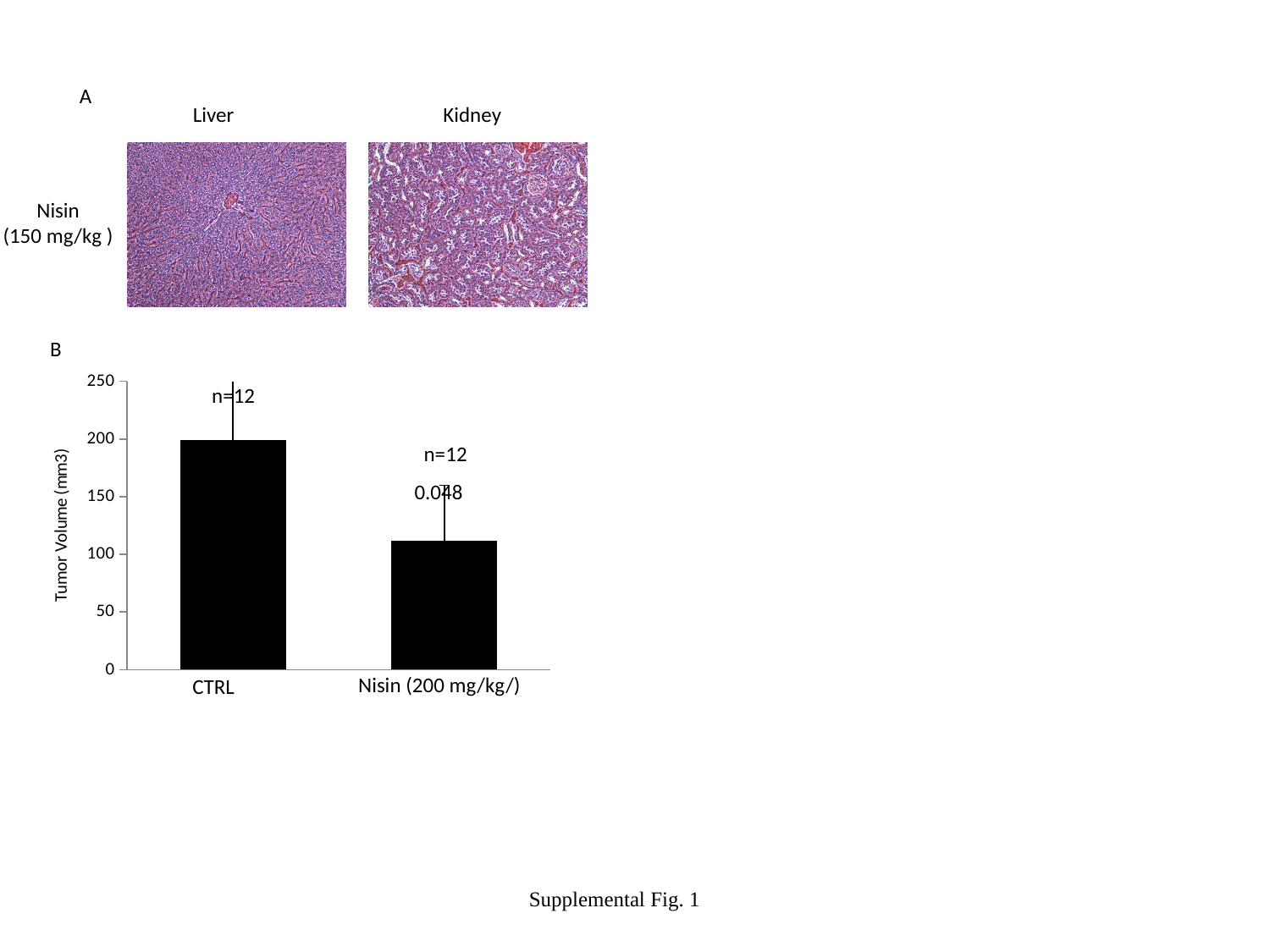

A
Liver
Kidney
Nisin
(150 mg/kg )
B
### Chart
| Category | Average |
|---|---|
| Control | 199.2145999999995 |
| Nisin | 112.1421 |n=12
n=12
0.048
Nisin (200 mg/kg/)
CTRL
Supplemental Fig. 1

## Slide 10
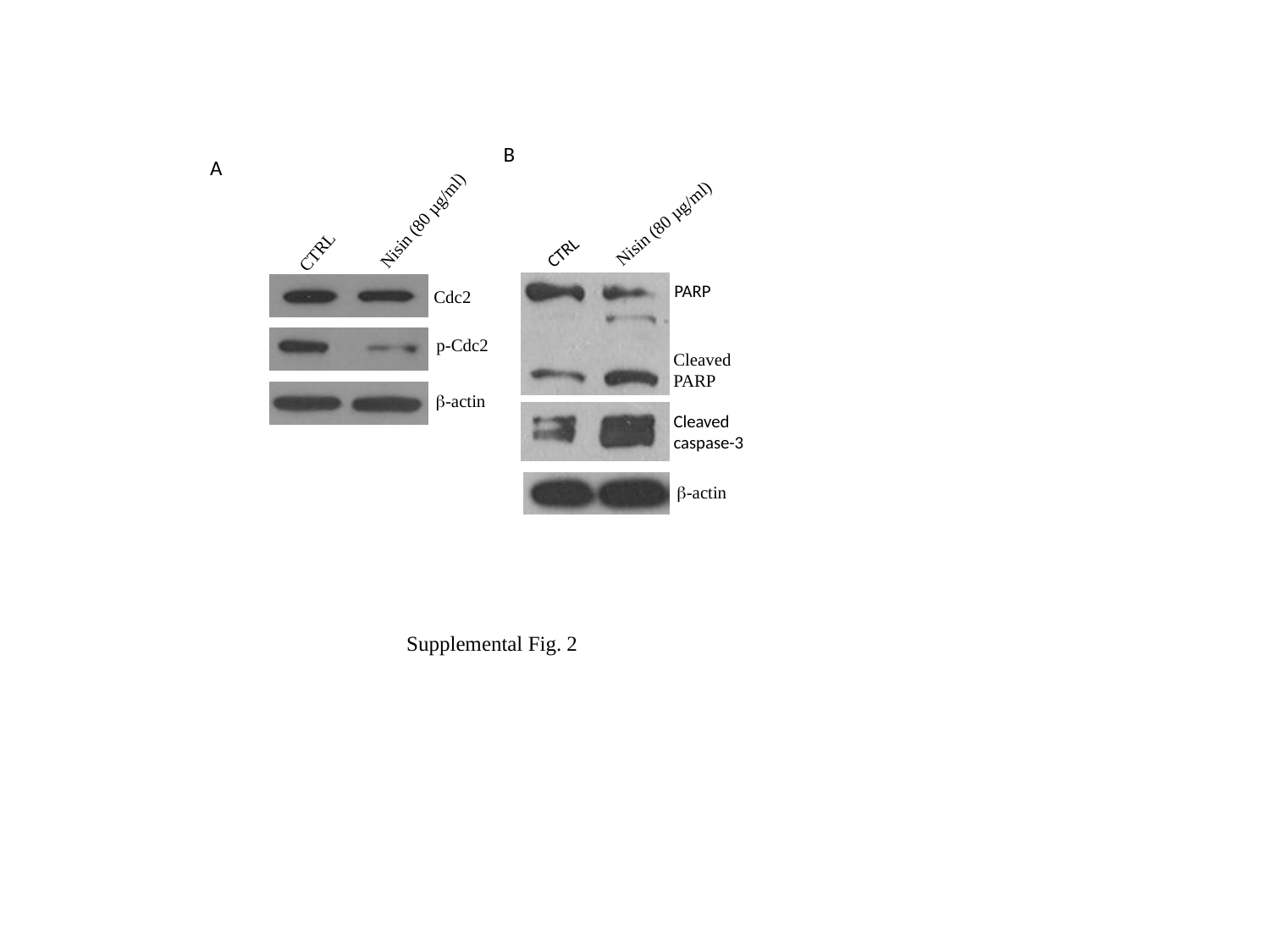

B
Nisin (80 µg/ml)
CTRL
Cdc2
p-Cdc2
b-actin
A
Nisin (80 µg/ml)
CTRL
PARP
Cleaved
caspase-3
b-actin
Cleaved
PARP
Supplemental Fig. 2
